# Canaries in a coal-mine? What the killings of journalists tell us about future repression

**DOI:** 10.1177/0022343316680859

**Published:** 2017-02-21

**Authors:** Anita R Gohdes, Sabine C Carey

**Affiliations:** Department of Political Science, University of Zurich; School of Social Sciences, University of Mannheim

**Keywords:** human rights, journalists, political violence, press freedom, repression

## Abstract

An independent press that is free from government censorship is regarded as instrumental to ensuring human rights protection. Yet governments across the globe often target journalists when their reports seem to offend them or contradict their policies. Can the government’s infringements of the rights of journalists tell us anything about its wider human rights agenda? The killing of a journalist is a sign of deteriorating respect for human rights. If a government orders the killing of a journalist, it is willing to use extreme measures to eliminate the threat posed by the uncontrolled flow of information. If non-state actors murder journalists, it reflects insecurity, which can lead to a backlash by the government, again triggering state-sponsored repression. To test the argument whether the killing of journalists is a precursor to increasing repression, we introduce a new global dataset on killings of journalists between 2002 and 2013 that uses three different sources that track such events across the world. The new data show that mostly local journalists are targeted and that in most cases the perpetrators remain unconfirmed. Particularly in countries with limited repression, human rights conditions are likely to deteriorate in the two years following the killing of a journalist. When journalists are killed, human rights conditions are unlikely to improve where standard models of human rights would expect an improvement. Our research underlines the importance of taking the treatment of journalists seriously, not only because failure to do so endangers their lives and limits our understanding of events on the ground, but also because their physical safety is an important precursor of more repression in the future.

## Introduction

**Figure 1.**
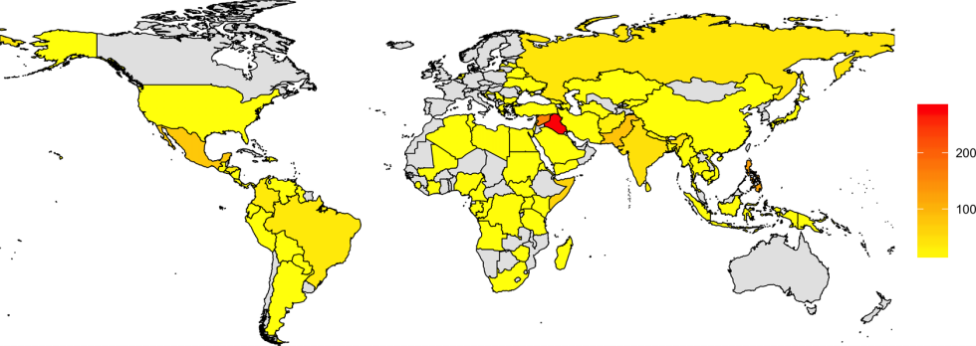
Number of journalists reported killed between 2002 and 2013

**Figure 2.**
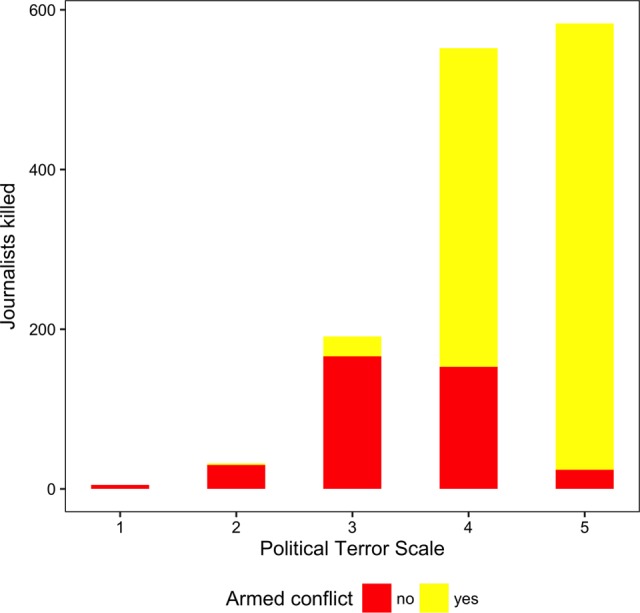
Number of journalists killed, by repression level, and whether there was an armed conflict in the same year (2002–13)

Governments across the globe often target journalists when reports and stories seem to offend them or contradict their policies. In 2015 alone, over 70 journalists were killed; most of them wrote about political issues.^1^ Figure 1 maps the killing of journalists between 2002 and 2013. The darker the shading, the more journalists were killed during that time period. Syria and Iraq are among the most dangerous places for journalists, with 162 and 287 journalists, respectively, reportedly killed between 2002 and 2013. But journalists are not only targeted in countries that experience a civil war, as in those two examples. Between 2002 and 2003, members of the press corps were killed in over 80 countries. To show that journalists are not only targeted in the most repressive countries, Figure 2 plots the number of journalists killed under varying overall human rights conditions. The x-axis represents the Political Terror Scale (PTS; Wood & Gibney, 2010), which captures the extent of physical integrity rights violations – the higher the value, the more repressive the regimes. In yellow, we highlight the killings of journalists that occurred in years of armed conflict, in red those that took place outside of armed conflict. Surprisingly, outside of armed conflict, journalists are mostly killed in countries where governments show at least some respect for human rights.

Journalists are frequently targeted for reporting uncomfortable news, not just during armed conflicts and not only in already repressive regimes. What does this tell us about the trajectory of the overall human rights situation? Can the killing of a journalist act as an indicator for subsequently increasing repression?

We suggest that the killing of journalists acts as a precursor for worsening state-sponsored repression – just as canaries in a coal-mine would be the first to signal distress if toxic gases had leaked into the mine and were polluting the air quality for miners. Independent journalists will be a thorn in the side of governments who are attempting to cover up violence, or who are trying to dominate the public narrative of why these measures are justified. The killing of a journalist provides us with information about the government’s willingness to use extreme measures to eliminate potential dissent and to remain in control. Journalists writing about organized crime and violence are also often targeted by those whose illegal activities are brought to light. In those instances, the killing of a journalists can signal spiraling violence, which often results in more repressive government behavior. In short, information about the killings of journalists should be a valuable indicator of human rights trajectories, highlighting which countries are at risk of deteriorating human rights. It can act as an early-warning signal for worsening repression, enabling policymakers to intervene or build resilience before violence has escalated.

The killing of journalists should be particularly useful as an early-warning signal in countries that show some, but not great, respect for physical integrity rights. For such countries it is difficult to tell whether they have ‘settled’ in this middle position, whether they are about to improve their respect for human rights, or whether they are on the verge of increasing repression. Stable and secure countries like Canada and Australia are unlikely to experience a sudden turn for the worse. Similarly, nobody expects immediate serious improvements of the bleak human rights conditions in countries embroiled in conflict and state-sponsored violence, such as in Syria or Sudan. While structural factors, such as economic development or democracy, can identify countries that are more likely to be at either end of the human rights scale, they are less suited for forecasting their short-term trajectory because structural characteristics change extremely slowly (see Chiba & Gleditsch, 2017). The killing of journalists reflects more dynamic changes that provide us with valuable insights on likely changes in human rights conditions in the following year, particularly for moderately repressive countries, for which the assessment of future human rights developments is inherently difficult.

To show that the killing of journalists can provide us with useful information about the trajectory of the overall human rights conditions in that country, we present a new global dataset on killings of journalists between 2002 and 2013.The dataset provides new insights into how many national or international journalists are targeted by different perpetrators across the years. It shows that international journalists are targeted only in rare circumstances, and that across all killings, the perpetrators usually remain unconfirmed. Our analysis shows that if a journalist is killed, state repression is significantly more likely to deteriorate in the following two years – particularly in countries that previously displayed low levels of repression. The killing of a journalist also highlights countries with continuing repression, where structural characteristics would suggest an improvement of human rights conditions. In the following, we summarize explanations for state-sponsored repression and elaborate why the killing of a journalist should help us assess the risk of worsening of human rights conditions.

## Assessing changes in government repression

Since the early global studies that analyzed why physical integrity rights are violated by government-related actors (see Mitchell & McCormick, 1988; Poe & Tate, 1994), research has made great progress in identifying characteristics that are associated with government repression. Scholars often utilize decisionmaking models to explain human rights violations. According to these models, governments weigh up the costs and benefits of repression, often comparing their own strengths with the perceived threat to their leadership (Poe, 2004). For example, the findings that democracies have better human rights records than other forms of political regimes is explained with democratic institutions increasing the cost of repressive behavior (Bueno de Mesquita et al., 2005).

Empirical studies on state repression often concentrate their theoretical arguments and empirical models on structural factors (for a summary, see Hill & Jones, 2014). Structural characteristics, such as regime type, development, and population size, are useful for separating countries into more or less likely human rights offenders, but are less suited for explaining developments *within* countries. They can usually tell us little about when and why governments suddenly *change* their strategy of torturing, disappearing or killing their own people, because these indicators change extremely slowly.

Another strand of research asks how the behavior of actors shapes government responses. The most consistent finding is that challenges to political authority in the form of civil war and violent dissent make governments respond with repression, labeled the ‘Law of Coercive Responsiveness’ (Davenport, 2007: 7). Yet exactly how dissent and repression affect each other is difficult to assess given their endogenous relationship. Ritter & Conrad (2016) suggest that if governments repress as a preventative measure, the state’s response to dissent is unclear. Although dissent should be particularly useful to assess the risk of repression because it varies across time, not only are data on dissent highly endogenous to repression, but reliable and valid data on dissent are hard to come by and are usually based on coding news sources (see Landman & Gohdes, 2013; Weidmann, 2016). Before we outline why data on journalist killings is likely to be reliable, we explain why the killing of journalists can act as a precursor for worsening repression.

## Government repression and the treatment of journalists

Press freedom is widely recognized as an important element of a well-functioning democracy. A free press ensures that political competition can take place and that the population is informed about the leaders’ decisions and behaviors, an essential element for holding rulers to account (Whitten-Woodring, 2009). Accountability is crucial for making democracies respect human rights; only democracies that have institutionalized effective accountability measures have better human rights records than other regimes (Bueno de Mesquita et al., 2005).

Leaders go to great lengths to avoid accountability, for example by outsourcing particularly heinous violence to irregular forces (Mitchell, Carey & Butler, 2014; Carey, Colaresi & Mitchell, 2015). Leaders who want to dominate the public narrative of their performance domestically, and who want to distort the representation of their rule internationally, are likely to have a strong incentives to interfere with press freedom. Whenever government actors arrange for a journalist to be killed, it is likely to feel under pressure and is concerned about losing control over the framing of certain issues. The killing of a journalist might be triggered by a story being uncovered that is unfavorable for the government. The killing would then result from unobserved earlier behavior that puts the government in a bad light. The killing of a journalist then picks up deteriorating security that leaves governments willing to order the murder, and can be the beginning of an overall deteriorating human rights situation.

The following case summarizes this mechanism. Ando Ratovonirina was a reporter and cameraman for a private broadcasting company in Madagascar. He was shot dead by presidential guards while covering antigovernment demonstrations in Antananarivo in February 2009. He was the first journalist killed on duty in Madagascar since the first recording of journalist deaths in 1992.^2^ In the following month, the mayor of Madagascar’s capital city proclaimed himself president (Bearak, 2009). The next year Amnesty International expressed serious concern about excessive violence used by the security forces and the extent of arbitrary imprisonment. In this example, the overall respect for human rights declined after the reporter was murdered.

Governments might also order the killings of journalists anticipating that they will carry out actions that are worth keeping hidden from public view. In this scenario the killing foreshadows government transgressions. Whether it is the urge to drastically intervene in how public debates are framed, or the desire to keep certain facts from coming to the surface, or whether it is the anticipation of having something to hide in the near future, killing a journalist reflects the government’s willingness and capability to use extreme measures to influence the flow of information to avoid accountability for illegal or very unpopular actions. Once governments have taken the step of ordering the killing of a journalist, over time they might grow less hesitant to apply repression more widely.

Maintaining plausible deniability can involve governments ordering the killing of a journalist in order to avoid being linked to the crime. For almost half of the journalists killed between 2002 and 2013, the perpetrator remains unconfirmed. Since governments have a motivation to hide their connection to the killing, and are likely to have the resources to do so, we assume that the majority of unconfirmed cases are linked to the government. The murder of a journalist, irrespective of whether the perpetrator can be linked to state forces or whether their identity remains unconfirmed, indicates that the government is feeling increasingly under pressure, forced to act, and willing and able to act violently, suggesting that they will become more repressive in subsequent years.

For example, in August 2013, Luis de Jesús Lima, a radio journalist working in Zacapo, Guatemala was gunned down outside his office.^3^ Lima was one of five members of the media killed in Guatemala in 2013. In the following year, respect for human rights declined, as police brutality and organized crime became increasingly common. In March 2015, another journalist, Danilo López, known for covering issues of corruption and the misuse of public funds, was also shot dead while on duty. His death goes hand in hand with increasing instability in Guatemala, painting a bleak picture for 2016 (see the *Economist*, 2015). We argue that this example reflects a wider trend, where the killing of a journalist signals the deterioration of overall human rights conditions.

*Empirical expectation 1:* The killing of a journalist by a government-sponsored or an unconfirmed actor is likely to signal subsequently deteriorating human rights conditions.

There are other causes of death for journalists. Some die in crossfire in ongoing conflicts, while others are killed by rebel groups or criminal gangs. During armed conflicts, rebels might have an incentive to murder journalists as they can interfere with their own preferred narrative of the nature and outcome of the conflict. The murder of a journalist by a criminal gang also reflects increasing insecurity. If journalists are killed by political opposition groups or criminal gangs, the state has lost the upper hand in controlling these violent groups, which increases the risk of more government-sponsored repression.

The death of Ahmed Rajib Haider presents an egregious example for such instances. On 15 February 2013 the Bangladeshi blogger was found dead in his own home; he had been hacked to death by religious fanatics with machetes. Haider had written a series of critical blog posts about extremist Islamist groups and Islamist fundamentalism in Bangladesh. Five perpetrators were arrested who confessed having received the order from Islami Chhatra Shibir, the militant student wing of Bangladesh’s Islamist organization Jamaat-e-Islami.^4^ One year later, Human Rights Watch warned that Bangladesh was ‘tumbling backwards’ on human rights, with increasing powers for the government, increased civil society restrictions, and abductions, killings, and arbitrary arrests by security forces becoming part of daily life (Human Rights Watch, 2014).

Crisis hot spots are also likely to attract journalists who aim to report about the events, putting them at greater risk of getting in the line of fire and indicating a deterioration of the safety in the country. During wars, governments often go to extremes to rein in violent groups. Valentino, Huth & Balch-Lindsay (2004) show that governments kill large numbers of civilians to drain the support base of the guerrillas. Violent political groups, such as guerrilla groups, are more threatening than non-political groups like criminal gangs. The killing of journalists by political non-state actors, such as rebel forces, can signal the increasing threat of such forces, triggering a harsh and quick response from the government. When criminal gangs kill journalists, governments might not immediately respond with increasing repression, but the increase in government violence might take place more slowly as their violence is less threatening than those of insurgencies. But in both cases we expect that the killing of a journalist by nongovernmental actors leads to increasing state repression.

*Empirical expectation 2:* The killing of a journalist by a nongovernmental actor is likely to signal subsequently deteriorating human rights conditions.

We expect our measure for killed journalists to be most useful as an early-warning indicator for increased repression in countries with currently limited government-sponsored violence. For regimes that already widely use torture, political imprisonment, extrajudicial killings, and disappearances, the killing of one (or more) journalist is unlikely to provide us with additional insights on subsequent levels of repression. Whitten-Woodring (2009) finds that in the most authoritarian regimes, media freedom is related to worse respect for human rights. Media freedom is linked to better human rights only in the most democratic regimes. We expect that worsening repression in the aftermath of a journalist being killed is only visible in countries where repression is not yet directed at the entire population. While we expect to see a general effect, the killing of a journalist should act as a precursor of increased repression primarily in countries with currently limited repression.

*Empirical expectation 3:* The killing of a journalist is likely to signal subsequently deteriorating human rights conditions, particularly in countries where repression is limited.

Under certain conditions, killings of journalists should *not* be linked to increasing repression. If a government kills journalists to cover up future repression, and if this strategy is successful, then we should *not* observe an increase in reported repression after the killing. While not all information flows about state violence depend on journalists, targeting journalists should restrict the reporting of future human rights violations, if successful – which would counteract any predictive value the killings of journalist might have. If killing journalists is a successful strategy in limiting the knowledge of or need for repression, then the recorded level of repression should *not* increase in the years following the killing of a journalist.

## A new global database on killed journalists

We present a new database that documents journalists who were reported to have been killed between 2002 and 2013. We focus on killings as the most reliable and valid indicator of violence committed against journalists. By definition, lethal violence can only take place once, whereas all other forms of violence such as imprisonment, torture, kidnapping, or intimidation can take place multiple times, and vary substantially in length and circumstances, making it harder to establish a definitive and comparable number of reported cases. Our data use three different sources that track such events across the world: the Committee to Protect Journalists (CPJ), the International Press Institute (IPI), and Reporters without Borders (RWB).

Our operational definition of journalists, based on the Committee to Protect Journalists, defines journalists as ‘people who cover news or comment on public affairs through any media – including in print, in photographs, on radio, on television, and online’.^5^ Our database includes associated personnel of media professionals, such as translators and administrative staff. The inclusive nature of this definition is particularly well suited given the time period we study: from 2002 communication via the Internet became an increasingly important part of news distribution, making blogging and other forms of online journalism increasingly influential and thus potentially threatening for governments.

The Committee to Protect Journalists (CPJ) is an independent non-profit organization that promotes press freedom, especially the right of journalists to work safely across the world. CPJ has full-time staff working in Africa, the Americas, Asia, Europe, Central Asia, the Middle East, and North Africa monitoring attacks on the press.^6^ Since 1992, CPJ has maintained a list of killed journalists across the world.^7^

The International Press Institute (IPI) is a global network of editors, media executives, and journalists committed to promoting press freedom and the safety of journalists. IPI was founded in 1950 in the aftermath of World War II and has members in over 120 countries. Since 1997, IPI has maintained a ‘Death Watch’ list, which records the names of journalists and media staff who were deliberately targeted in their role as members of the press.^8^

Reporters without Borders (RWB) is a non-profit organization, founded in 1985, that aims to promote the safety of journalists, particularly by monitoring attacks on press freedom, fighting against censorship, and providing support to journalists working in dangerous environments. RWB counts correspondents in 150 countries and has been reporting the names of journalists killed across the world since 2002 as part of their Press Freedom Barometer.^9^ RWB only includes cases in their database where the motive of the killing was clearly established.

We consider the information collected by these groups to be superior to information collected through news sources alone. All three groups act as global interest groups for journalists and count members of the media as part of their teams. Since all groups provide information on each victim’s name, date, and country of death, we match every record of a journalist killed across the three lists. Matching was done by hand. All records were compared to each other and determined to be a match or not. We limit our dataset to the years from 2002 to 2013, the period for which we have data from all three sources. The matched records provide us with the de-duplicated number of identified journalists killed between 2002 and 2013.

### Coding the perpetrators

We use auxiliary information collected by the three groups to establish perpetrator identities. This is no easy undertaking, as killing members of the press corps violates international law. Governments will generally have few incentives to broadcast the silencing of media personnel to the outside world. Furthermore, governments will usually be in a superior position to cover their tracks and to deny responsibility or involvement in the murder of journalists. Therefore, for a large number of killed journalists the perpetrator was not clearly identifiable. In contrast to governments, nongovernmental groups might try to gain international attention by claiming responsibility for the killing of journalists. For example, the radical extremist Islamic State claimed to have beheaded US journalist James Foley in August 2014, and al-Qaeda in Yemen claimed responsibility for the Charlie Hebdo attack in France in January 2015.

If the killing of a journalist can be linked to officials of the government, military, the police, pro-government militias, paramilitary groups or troops, national guards or death squads, intelligence or security agents, or international forces working for the government, the perpetrator is coded as belonging to the *State*. If a journalist was killed by a political group that was not part of the government, by a rebel, religious, or extremist group, by antigovernment militants or tribal groups, the perpetrator is coded as being a *Non-state political group*. Killings committed by criminals, mobs, drug gangs or influential families are coded as *Non-political actors*. Some journalists in the database died of other causes, such as natural disasters, diseases or in accidents. These are coded as *Accidents*. Accidental deaths are excluded from all analyses. All other recorded killings are coded as *Perpetrator unconfirmed*.

## Journalist killings and changes in state repression

To measure state repression we turn to the widely used Political Terror Scale (PTS; Wood & Gibney, 2010). PTS includes two categorical measures of physical integrity rights that range from 1 to 5. The scale is based on information from the US State Department’s yearly Human Rights reports and Amnesty International’s yearly country reports (Wood & Gibney, 2010).^10^ The PTS distinguishes between countries (1) under secure rule of law, (2) with limited amount of imprisonment for nonviolent political activity, (3) where extensive political imprisonment and political murders are common, (4) with large numbers of murder, imprisonment, and disappearances, and (5) with terror expanded to the whole population (see Poe, Carey & Vazquez, 2001: 658).

Changes in human rights are generally difficult to explain because the level of observable repression changes only very slowly; the extent of repression at time *t* is thus highly dependent on the level of repression at time *t*–1 (Carey, 2010). This is not only because governments change their behavior very slowly, but also because common human rights measures capture broad and therefore relatively stable categories. For a country to move from one category to another of the Political Terror Scale (Wood & Gibney, 2010), substantial changes in human rights conditions are necessary.

While using a broad human rights measure makes detecting and explaining changes more difficult, it avoids potential problems of endogeneity, where changes in our main indicator, the killing of journalists, could automatically trigger changes in the recorded level of repression. Our measure of killed journalists is less dependent on changes in media reports than measures that attempt to count instances of repression, such as data on one-sided killings, for example (see Weidmann, 2016).^11^ If the killing of a journalist automatically led coders to put the country in a worse human rights category, the results would be driven by the coding procedures. Given the broad nature of the PTS categories, it is highly unlikely that the killing of a journalist leads to a change in the coding of the human rights condition. As shown below, the overwhelming majority of journalists killed work for locally owned news outlets and the perpetrators remain unconfirmed. These cases receive little attention, and due to lack of evidence, governments are not held accountable. To further account for potential problems of endogeneity, we model the relationship between journalist killings in the previous year and repression in the following year, and look at two, three, four, and five-year lags of journalists killed.

**Figure 3.**
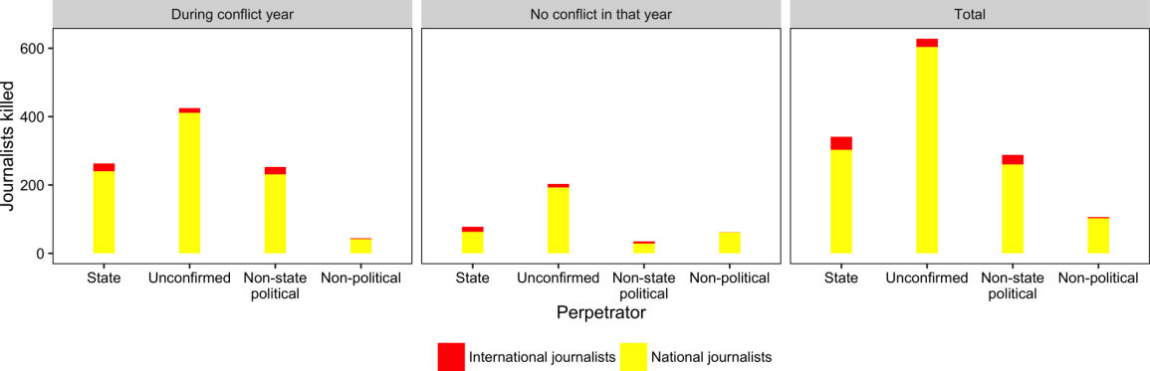
Journalists killed in and outside of conflict years, 2002–13

The right panel in Figure 3 reveals that the overwhelming majority of all journalists killed between 2002 and 2013 were working in their home country. Excluding accidents, 93% of all journalists killed were working locally; only 7% of all journalists killed were working in a foreign country. Since we do not identify the country of residence of the foreign journalist, the count also includes instances where the killed journalist was based in a neighboring country. Hence, not all international journalists represent large international media outlets. The distinction between national and international journalists helps us understand what the potential effects on the information environment are in the aftermath of a member of the press being killed. While the killing of prominent international journalists is usually met by international outrage, the killing of local journalists generally draws far less attention, particularly where perpetrators remain unconfirmed.

The left and middle panel of Figure 3 distinguish between journalists who were killed during an armed conflict and those outside of conflict years (as defined by Melander, Pettersson & Themnér, 2016). As Figure 2 already showed, more journalists are killed during armed conflict years, and the level of repression during these years is very high.

However, a substantial number of journalists are also killed by state and unconfirmed perpetrators outside of conflict. Unsurprisingly, more journalists are killed by non-state political actors during armed conflicts than during peace years. In contrast, a larger number of journalists are killed by non-political actors during peace times.

The graph in Figure 3 demonstrates that the patterns of government targeting of journalists (including those with unconfirmed perpetrators) look almost the same – albeit at a different scale – during and outside of armed conflict. A difference can be found in the number of journalists killed by non-state political groups. Armed groups such as rebels are more likely to attack journalists in the midst of armed conflict than otherwise. Absent armed conflict, very few international journalists are targeted by non-state political groups.

Governments rarely target foreign journalists in times of relative peace. Since only few killed journalists are foreign, the risk is very small that the killing of journalists would increase international attention and therefore lead to more detailed human rights reports, which might result in coding the country as more repressive. In the majority of cases, it is local journalists who are target and killed.

## Multivariate analysis

To investigate whether the killing of a journalist can tell us something about the overall subsequent human rights situation, we conduct a series of multivariate analyses controlling for the most common predictors of state repression identified in the literature (see Hill & Jones, 2014). Our sample includes 166 countries between 2003 and 2014 for which information on state repression is available and which have a population above 500,000.

Our principal variable of interest is the number (*Count*) of journalists killed in the previous year.^12^ We also test whether killed journalists signal the deterioration of human rights in the following two, three, four, or five years. Additionally, we construct a categorical variable with specific *Categories* that measures whether zero, one to four, five to nine, or ten or more journalists were killed in the previous year.

Internal dissent consistently predicts increasing state repression (Carey, 2010). We use the UCDP/PRIO armed conflict data (Melander, Pettersson & Themnér, 2016) and include a binary indicator that takes on the value 1 if the government was involved in any organized armed confrontation that resulted in at least 25 battle-related deaths (UCDP/PRIO, 2014: 9) and 0 otherwise. To account for possible changes in repression due to institutional configurations, we include the revised Polity scores as a measure for how democratic a country was in any given year (Marshall & Jaggers, 2001). The Polity project recommends recoding countries classified as going through ‘interregnum’ periods as anocracies (with a value of 0), while Gleditsch & Ruggeri (2010) suggest that they are more accurately represented as highly autocratic (with a value of –10). We test our models using both operationalizations. To account for size and wealth of a country, we include the natural log of population size, as well as the natural log of the real gross domestic product per capita (World Bank, 2015). With the exception of the armed conflict indicator, we lag all control variables by one year. We include *k*–1 binary variables that indicate the previous year’s repression level in each country, where *k* is the number of possible categories.

Lastly, we test our third hypothesis by interacting our journalist count variable with the binary variables that indicate whether previous year’s repression was either at PTS level 2 or 3.^13^

### How does repression change after the killing of journalists?

We use ordered probit estimation to analyze how likely it is that the level of repression changes in the year after a journalist was killed. We commence by including one- and two-year lags of the number of journalists killed. Table I presents the results. Comparing the first and the second models, we see that including the number of journalists killed improves the model fit, reducing both AIC and BIC values. The model in the second column provides a general confirmation of our argument: the number of journalists killed in the previous two years is positively and significantly associated with higher levels of repression. We thus find overall support that killings of journalists are associated with higher levels of repression in the future. The remaining control variables show the expected effects: higher levels of democracy and economic development are associated with lower levels of repression, while armed conflict and population size are significantly correlated with increased repression. The thresholds refer to the estimated cutpoints of the probit model for the different levels of the dependent variable.

**Table I.** Number of journalists killed, by perpetrator

	*Baseline*	*All killed*	*State*	*State + unconfirmed*	*Unconfirmed*	*Pol. group*	*Non-pol. group*	*Interaction*
All killed (*t*–1)		0.04†						0.01
		(0.02)						(0.02)
All killed (*t–*2)		0.06**						0.06**
		(0.02)						(0.02)
			0.01					
			(0.03)					
State perp (*t–*2)			0.06*					
			(0.03)					
State + unconfirmed (*t–*1)				0.04†				
				(0.02)				
State + unconfirmed (*t–*2)				0.07**				
				(0.02)				
Unconfirmed (*t–*1)					0.09*			
					(0.05)			
Unconfirmed (*t–*2)					0.09†			
					(0.05)			
Pol. group (*t–*1)						0.30**		
						(0.11)		
Pol. group (*t–*2)						0.11		
						(0.11)		
Non-pol. group (*t–*1)							0.11	
							(0.11)	
Non-pol. group (*t–*2)							0.21†	
							(0.11)	
All killed (*t–*1)*LDV=2								0.45*
								(0.17)
All killed (*t–*1)*LDV=3								0.13**
								(0.05)
LDV = 2	1.88***	1.87***	1.88***	1.87***	1.87***	1.88***	1.87***	1.85***
	(0.11)	(0.11)	(0.11)	(0.11)	(0.11)	(0.11)	(0.11)	(0.11)
LDV = 3	3.39***	3.35***	3.38***	3.36***	3.35***	3.39***	3.37***	3.35***
	(0.14)	(0.14)	(0.14)	(0.14)	(0.14)	(0.14)	(0.14)	(0.14)
LDV = 4	5 14***	5.03***	5.11***	5.05***	5.05***	5.10***	5.09***	5.11***
	(0.17)	(0.17)	(0.17)	(0.17)	(0.17)	(0.17)	(0.17)	(0.18)
LDV = 5	6.99***	6.87***	6.95***	6.88***	6.90***	6.93***	6.97***	6.94***
	(0.26)	(0.27)	(0.26)	(0.27)	(0.27)	(0.27)	(0.26)	(0.27)
Polity2	−0.03***	−0.04***	−0.03***	−0.04***	−0.04***	−0.03***	−0.03***	−0.04***
	(0.01)	(0.01)	(0.01)	(0.01)	(0.01)	(0.01)	(0.01)	(0.01)
Log pop.	0.15***	0.14***	0.15***	0.14***	0.14***	0.15***	0.14***	0.14***
	(0.02)	(0.02)	(0.02)	(0.02)	(0.02)	(0.02)	(0.02)	(0.02)
Log GDP p.c.	−0.21***	−0.22***	−0.21***	−0.22***	−0.22***	− 0.21***	−0.21***	−0.22***
	(0.02)	(0.02)	(0.02)	(0.02)	(0.02)	(0.02)	(0.02)	(0.02)
Armed conflict	0 74***	0.70***	0.72***	0 70***	0.72***	0.69***	0.76***	0.71***
	(0.10)	(0.10)	(0.10)	(0.10)	(0.10)	(0.11)	(0.10)	(0.10)
Threshold 1/2	0.75†	0.44	0.71†	0.51	0.41	0.65†	0.52	0.37
	(0.38)	(0.39)	(0.38)	(0.39)	(0.39)	(0.38)	(0.39)	(0.39)
Threshold 2/3	2.96***	2.66***	2.93***	2 74***	2.64***	2.86***	2.74***	2.62***
	(0.39)	(0.39)	(0.39)	(0.39)	(0.39)	(0.39)	(0.39)	(0.39)
Threshold 3/4	5.39***	5.10***	5.36***	5.17***	5.08***	5.29***	5.17***	5.07***
	(0.40)	(0.41)	(0.40)	(0.40)	(0.41)	(0.40)	(0.41)	(0.41)
Threshold 4/5	7.88***	7.64***	7.86***	7.70***	7.61***	7.84***	7.68***	7.60***
	(0.42)	(0.43)	(0.42)	(0.43)	(0.43)	(0.42)	(0.43)	(0.43)
AIC	2,531.89	2,514.82	2,531.11	2,519.08	2,515.72	2,520.83	2,526.80	2,505.53
BIC	2,598.30	2,592.29	2,608.58	2,596.56	2,593.19	2,598.30	2,604.27	2,594.07
Log Likelihood	–1,253.94	–1,243.41	–1,251.56	–1,245.54	–1,243.86	–1,246.41	–1,249.40	–1,236.77
No. obs.	1,871	1,870	1,870	1,870	1,870	1,870	1,870	1,870

***p < 0.001, **p < 0.01, *p < 0.05, †p < 0.1. Ordered probit regression.

Distinguishing between different perpetrators reveals that state-perpetrated killings of journalists today are a significant indicator for deteriorating human rights respect two years later. The two-year lag confirms the assumption that levels of repression generally change slowly, and that violence committed against the press corps will be an early precursor of worsening human rights conditions.

Since we expect the majority of unconfirmed killings to be perpetrated by forces loyal to the government, we group these cases together in the next model, and indeed find that state and unconfirmed killings are not only significantly associated with higher levels of repression two years into the future, but also already in the following year. When only looking at unconfirmed cases, the results look quite similar. The combined evidence of these models supports our first hypothesis that killings committed by the state or unconfirmed perpetrators are likely to signal subsequently deteriorating human rights conditions.

Moving to the killings committed by political groups not affiliated with the government, we find a more immediate relationship than with the state-related killings. The model shows that where political groups kill journalists, the government is significantly more likely to increase its level of repression already in the following year. This result supports our second hypothesis and suggests that governments increase repression fairly quickly if political actors create insecurity that manifests itself in the killing of journalists. The last perpetrator group in our database is the non-political actors, such as criminal groups or gangs. Here we see a more delayed relationship again, where killings perpetrated by such groups are likely to be associated with increased government violence two years later. Killings by non-political groups do not trigger an equally quick response from the government as killings perpetrated by political groups, such as insurgents. Instability due to crime is likely to be seen as less threatening than instability resulting from armed political groups, which could explain the different time lags with which governments respond with increased repression.

The final model aggregates all killings into one indicator and interacts this count variable with the binary variables that measure whether the previous levels of repression were either 2 or 3.^14^ Both interaction coefficients are positive and significant, offering support for the third hypothesis, stating that the killing of journalists is a particularly prominent signal of worsening human rights in countries where repression is limited. Comparing goodness of fit over all models in Table II, the last model, accounting for different levels repression in the previous year, displays the best fit. Overall, the results show that models including an aggregated measure of journalists killed (regardless of perpetrator) display a better fit.

**Table II.** Different thresholds of number of journalists killed and repression in the following year

	All killed (thresholds, *t–*1)	All killed (thresholds, *t–*2)
1–4 killed (*t–*1)	0.34***	
	(0.09)	
5–9 killed (*t–*1)	0.71**	
	(0.23)	
>=10 killed (*t–*1)	1.05**	
	(0.35)	
1–4 killed (*t–*2)		0.38***
		(0.10)
5–9 killed (*t–*2)		0.74**
		(0.24)
>=10 killed (*t–*2)		1.07**
		(0.38)
LDV = 2	1.86***	1.85***
	(0.11)	(0.11)
LDV = 3	3.33***	3.32***
	(0.14)	(0.14)
LDV = 4	4.99***	5.01***
	(0.18)	(0.18)
LDV = 5	6.83***	6.83***
	(0.27)	(0.27)
Polity2	−0.04***	−0.04***
	(0.01)	(0.01)
Log pop.	0.14***	0.14***
	(0.02)	(0.02)
Log GDP p.c.	−0.22***	−0.22***
	(0.02)	(0.02)
Armed conflict	0.71***	0.71***
	(0.10)	(0.10)
Threshold 1/2	0.33	0.32
	(0.39)	(0.39)
Threshold 2/3	2.57***	2.56***
	(0.39)	(0.39)
Threshold 3/4	5.01***	5.01***
	(0.41)	(0.41)
Threshold 4/5	7.55***	7.54***
	(0.43)	(0.43)
AIC	2,511.93	2,509.13
BIC	2,594.94	2,592.13
Log likelihood	–1,240.96	–1,239.56
No. obs.	1,871	1,870

***p < 0.001, **p < 0.01, *p < 0.05, †*p* < 0.1. Ordered probit regression.

Building on these results, we rely on the aggregated measure to construct a categorical variable with specific *Categories* that measures whether zero, one to four, five to nine, or ten or more journalists were killed in the previous year. Table II reports the results when including the categories as factorial variables, where the reference category captures when no journalist was previously killed. The first model includes the category variable as a one-year lag, and the second includes the two-year lag. The results show that compared to observations where no journalists were killed, all else equal, all categories are statistically significantly associated with higher levels of repression. Unsurprisingly, the higher the category (i.e. number of journalists killed) the larger the coefficient and thus the more substantial the effect is. The second model further confirms that two years after a journalist is killed, and regardless of perpetrator, levels of repression are very likely to increase.

Next, we simulate predicted probabilities using the regression parameters of the category model with the one-year lag, and apply them to two hypothetical observations where all control variables are held constant at their mean or modal value, but in one case no journalists were killed in the previous year, while in the other case a certain number of killings occurred.

**Figure 4.**
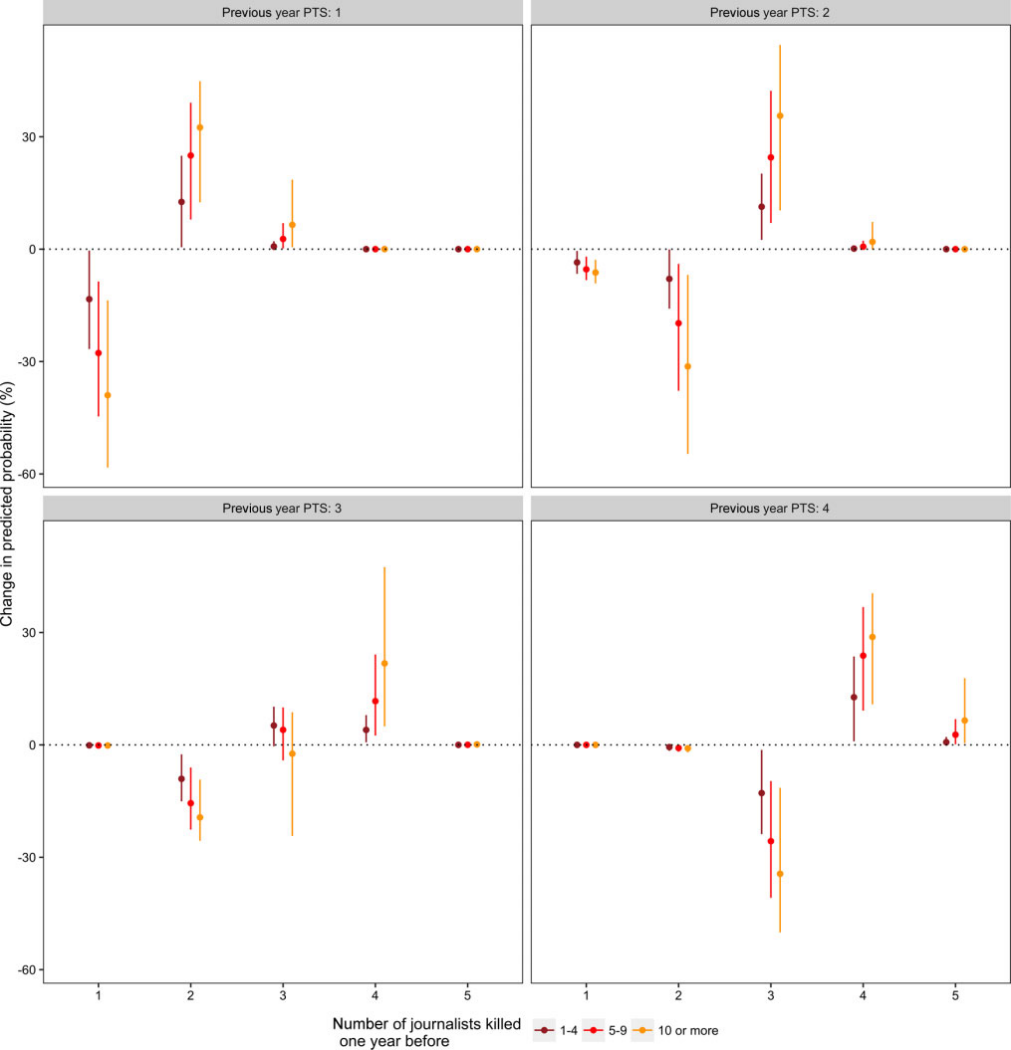
Changes in predicted probabilities when moving from no journalists to 1–4, 5–9, and 10 or more journalists killed in the year before

Figure 4 plots the changes in the simulated predicted probability of each repression level, given different levels of repression in the previous year. The lines denote the 2.5% and 97.5% quantiles. The dark red line plots the change in predicted probability when going from zero to one to four journalists killed, the light red line plots the effects going from zero to five to nine journalist killed, and the yellow line represents the model going from zero to ten or more journalists killed in the previous year.

The top left panel shows the changes in predicted probabilities of different repression levels in a country without physical integrity rights violations in the previous year (PTS at (*t*–1)=1). If between one and four journalists are killed, the probability of this country maintaining a clean human rights record in the following year drops by about 15 percentage points. Conversely, the predicted probability of such a country becoming more repressive (moving from PTS=1 to PTS=2) increases by about 15 percentage points. The changes become more pronounced when between five and nine journalists are killed, and even more extreme when ten or more journalists are killed. The top right panel shows a similar picture, this time for a country with limited repression (PTS at (*t*–1)=2). In the event of a low number of journalists being killed (between one and four), the probability of moving into a higher category of repression (PTS=3) increases by about 10 percentage points. Again, the changes in predicted probabilities are substantially larger the more journalists are killed, irrespective of perpetrator.

The upper panels offer important evidence supporting our argument: the killings of journalists seem to be a pertinent precursor of a deteriorating human rights situation in countries where citizens previously enjoyed relatively high levels of protection. Regardless of who the perpetrator is, killings of journalist are associated with higher levels of repression in the following year.

The lower two panels of Figure 4 show the changes in predicted probabilities in countries that already exercised considerable levels of repression. To recall, a score of 3 on the Political Terror Scale means that governments commonly torture, execute, and imprison people for political reasons and that dissidents are frequently held in detention for unlimited periods of time. One might assume that the killing of journalists should not be associated with equally visible changes in repression in countries that *already* practice frequent repression. The lower left panel shows that while the killing of a journalist in the previous year does not significantly decrease the probability of a country maintaining its current level of repression, it substantially and significantly decreases its chances of *improving* its human rights score, and significantly increases the chances of this country becoming more repressive. Similarly, countries that already saw high levels of repression against dissidents, opposition groups, and other politically dangerous elites in the previous year (PTS at (*t*–1)=4) are highly unlikely to improve their human rights conditions if a journalist was killed at (*t*–1).

The results suggest that where human rights are generally respected and opposition leaders can speak their mind without running the risk of being imprisoned, disappeared, or executed, the killing of journalists can signal a dangerous shift in the government’s willingness to use force against those who challenge their political authority. Where repression is already frequently used against perceived opponents, the killing of a journalist is a viable indicator that there will be no improvement in this situation in the near future.

### Predictions

We turn to testing the predictive power of our model in two steps. First, we perform one detailed out-of-sample prediction, where we look at individual cases that can be correctly predicted when including information on journalists killed.^15^ In a second step we compare the overall accuracy of the different empirical models presented here through the rank probability score, similar to Daxecker & Prins (2017).

We divide the full sample of observations into a training set and a test set. The training set includes all observation through to the year 2011, and the test set consists of the last three years of the dataset (2012–14). We estimate each model using the training set, and then use the parameters to predict the levels of repression for the years 2012–14. We compare the baseline model and the last model in Table I. The number of correctly predicted outcomes as well as the rank probability score are presented as a measure of accuracy of the predictions.

Table III compares the number of correctly predicted outcomes made by the baseline model and the model including information on killed journalists. Including information on journalist killings adds five correctly predicted outcomes. Table IV lists the observations where the baseline model predicted incorrect outcomes, but that were correctly predicted by the journalist model. In both Iraq and Sierra Leone repression increased in 2012. In both cases the baseline model predicts no change in repression, but drawing on information about journalists killed in previous years, the journalist model is able to predict these correctly. Interestingly, the baseline predicts a worsening human rights situation in Malaysia in 2013, but the journalist model correctly predicts a consistently high human rights record. The remaining cases confirm the pattern visible in Figure 4: where repression is already comparably high, the killing of journalists will not necessarily signal an increase in repression, but it will certainly *not* predict an improvement in human rights respect, even when other important variables (such as democratic institutions, economic development, or the absence of armed conflict) might predict improvements. In all five cases, the baseline model predicts human rights improvements, whereas the journalist model correctly predicts consistently high levels of repression.

**Table III.** Number of correct/false predictions made with and without inclusion of information on journalists killed in previous years

*Prediction*	*Journalists*	*Baseline*
Wrong	110	115
Correct	361	356

**Table IV.** Improved predictions through inclusion of information on journalists killed in previous years

*Year*	*Country*	*Lag_PTS*	*PTS*	*Baseline*
2012	Iraq	3	4	3
2012	Sierra Leone	2	3	2
2013	Libya	4	4	3
2013	Malaysia	2	2	3
2013	Mexico	4	4	3
2014	Mexico	4	4	3
2014	Pakistan	5	5	4

Lastly, we calculate the rank probability scores as a measure of accuracy for different model specifications including the killing of journalists, and compare them to the baseline model. For this we make out-of-sample predictions for each model following the same procedure as above, and calculate the rank probability score for each case. The rank probability score is a measure of accuracy of probability forecasts that is useful when forecasting more than two categories, as with different levels of repression (measured with the PTS). Instead of only comparing the number of correctly predicted outcomes, the rank probability scores evaluate the difference between probabilities produced by the forecast with the observations (see e.g. Brandt, Freeman & Schrodt, 2014: 948). Both higher probabilities assigned to the correct outcome category and a sharper distribution of probabilities over the different categories will lead to a better rank probability score, thus indicating a higher accuracy of the forecast.^16^

**Figure 5.**
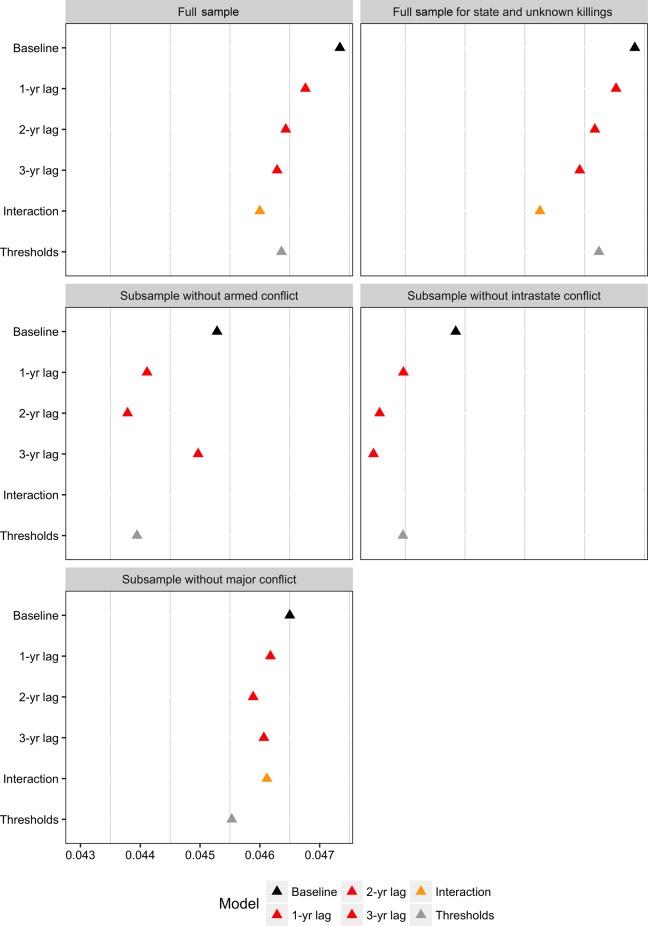
Rank probability scores for different models

Due to the big differences in the number of journalists killed within and outside of armed conflicts, we run our out-of-sample predictions for different subsets of our data. Figure 5 plots the different rank probability scores for each model and for different subsets. The top left panel shows the scores for the full sample using models including all journalist killings, whereas the top right panel plots the scores for the models that only include journalists killed by the state and unconfirmed perpetrators. The left middle panel shows the accuracy of the predictions of models for the subsample of country-years where no armed conflict took place. The right middle panel’s subsample only excludes intrastate conflicts, and the bottom panel shows the models trained and predicted on a sample excluding major conflicts with over 1,000 battle-deaths.

Across all panels, the baseline model (in black) has the highest rank probability scores, indicating that it has the lowest level of accuracy. Adding information on journalists killed thus improves the accuracy of the predictions. For the full sample models, the interaction model (in yellow) performs best, which is not surprising, given that it is also the model with the best goodness of fit statistics. The two middle panels excluding armed and intrastate conflict do not have scores for the interaction models as their inclusion overfits the model. The scores here indicate that in ‘peaceful’ years, the models including multiple lags (all in red) of the journalist variable provide the best accuracy – evidently killings that occurred further in the past still function as important signals for future repression. Keeping in mind the small scale of the x-axis, we can conclude that while the improvements of accuracy are substantially not very large, they are consistent across different model specifications and subsamples of the data. Including information on journalists killed leads to previously unexpected insights on otherwise unforeseeable levels of repression in the future. Furthermore, our models also show that when journalists are killed, human rights conditions are unlikely to improve where standard models of human rights would have predicted an improvement.

## Conclusion

We sought to understand whether the killing of journalists is a precursor for deteriorating human rights conditions. Independent journalists will be a thorn in the side of governments who face internal unrest and fail to subdue it without resorting to further repressive means, such as political imprisonments and murder. Silencing critical voices in the media also plays into governments’ attempts to dominate the public narrative of why repressive measures against political opponents are justified. Using new data on the killing of journalists, our analysis shows that where a journalist was killed, repression was significantly more likely to increase in the following two years. Despite the difficulties in using a very specific event, the killing of a (single) journalist, to assess changes in very static characteristics, the violation of physical integrity rights, our new measure can help us to pinpoint countries that might otherwise have not been on our radar for deteriorating human rights respect. We find that regardless of the perpetrator, the number of journalists killed in a country is a useful indicator of future repression, and the relationship is most pronounced in countries that currently experience only limited forms of state-sponsored repression, for which subsequent levels of repression are usually hard to predict. Our findings emphasize the policy relevance of carefully observing the treatment of journalists in countries with relatively good human rights records. Our results are instructive for circumstances where targeted and measured responses from the international community are most likely to successfully prevent further escalation of violence.

Our new dataset on journalists killed between 2002 and 2013 shows that journalists are often targeted outside of armed conflict. It also highlights that foreign journalists are killed comparatively rarely and that for most killings of a (local) journalist the perpetrator cannot be confirmed. The killing of a journalist is usually carried out in a way that attracts either minimal attention or cannot be traced back to whoever ordered the killing. It suggests that governments resort to arranging the killing of local journalists to control or limit public debate and the flow of information, while minimizing the risk of being linked to these crimes.

The findings also suggest that killing a journalist will not prevent a government from being subject to some international scrutiny. If murdering a member of the press was a successful tool in avoiding information about human rights violations becoming known, then we would not be able to observe any link between the killing of a journalist and the human rights classification of that country. It also highlights the important work of organizations like Amnesty International and the US State Department in uncovering instances of human rights violations, providing alternative sources of information that are not directly linked to the media.

Our results point to important questions for which we currently have few systematic answers. For example, while our new data tell us that few foreign journalists are killed because of their profession, we do not know how many are deported or how many are prohibited from entering a country in the first place. Both of these aspects are likely to provide us with insights into the intentions of a government to avoid visibility and (international) accountability – and to use more violence against its own population.

What might our study tell us about human rights conditions one year from now? According to the Committee to Protect Journalists, over 70 journalists were murdered in 2015. For example, four journalists were killed in Bangladesh between January and October 2015. Three of those were local bloggers, known for their critical stance against radical Islamists, the fourth a naturalized US citizen of Bangladeshi origin. While the investigation of this latter journalist attracted the help of the FBI, the others received little international attention. One of the bloggers, Ananta Bijoy Das, was murdered by unidentified perpetrators the day after he criticized the police on Facebook for their investigation of the murder of two other bloggers. While the killers of the blogger Washiqur Rahman Babu were captured, they had apparently no knowledge of the activities of the blogger and reported to have been ordered to kill this person. The killing of bloggers by unidentified perpetrators does not bode well for the overall human rights conditions in Bangladesh in 2016. Other countries with murdered journalists in 2015 include Brazil, Mexico, Turkey, Ukraine, and Kenya.^17^

Killing members of the media is in and of itself an egregious crime that demonstrates an utter failure to respect the importance of an independent and free press. Every mistreatment of journalists is a serious violation of the basic right to freedom of speech. Our analysis highlights that it should also send out warning signals for possible future and more widespread repression.

## Supplementary Material

Supplementary material

Supplementary material

Supplementary material

Supplementary material

Supplementary material

Supplementary material

Supplementary material

Supplementary material

Supplementary material

## Notes

1 See Committee to Protect Journalists: https://www.cpj.org/killed/2015/ (accessed 23 February 2016).

2 See report by Committee to Protect Journalists at https://cpj.org/killed/2009/ando-ratovonirina.php.

3 See the Committee to Protect Journalists at https://www.cpj.org/killed/2013/luis-de-jesus-lima.php, accessed 5 October 2015.

4 See https://cpj.org/killed/2013/ahmed-rajib-haider-1.php.

5 See https://cpj.org/killed/methodology.php.

6 See https://cpj.org/about/faq.php.

7 See https://cpj.org/killed/methodology.php, for their methodology and distinction between confirmed and unconfirmed killings. From 2003 onwards, CPJ includes the deaths of media support workers (i.e. translators and administrative staff) and we include this information in our database as well. We include confirmed and unconfirmed killings in our database, but we differentiate between death by accident and deaths perpetrated by other individuals.

8 See http://ipi.freemedia.at/death-watch.html.

9 See https://rsf.org/en/barometer.

10 We rely on the measure based on the US State Department, but where this information is missing (for example for the USA), we impute it with the measure by Amnesty International.

11 As our study only covers recent years (after 11 September 2001), we expect the effect of changes in the level of latent repression associated with different PTS categories to be negligible (Fariss, 2014).

12 Our analysis commences in 2003, as the first indicator for killed journalists is available for 2002, and includes 2014, where we look at the effect of journalists killed in 2013.

13 Including more than two interaction terms at a time leads to an overidentified model.

14 Further model specifications including interactions with previous levels of repression can be found in the Online appendix.

15 See Ward & Beger (2017) on the benefits of out-of-sample prediction.

16 The RPS was calculated using the verification package in R. The scores rank from 0 to 1 where lower values indicate higher accuracy.

17 For details on these cases, see the Committee to Protect Journalists at https://www.cpj.org/killed/2015/, accessed 23 February 2016.
